# The Association Between BMI and Inpatient Mortality Outcomes in Older Adults With COVID-19

**DOI:** 10.7759/cureus.11183

**Published:** 2020-10-26

**Authors:** Akwe Nyabera, Sofia Lakhdar, Matthew Li, Theo Trandafirescu, Sakina Ouedraogo Tall

**Affiliations:** 1 Internal Medicine, Icahn School of Medicine at Mount Sinai, Queens Hospital Center, New York, USA; 2 Pharmacy, Icahn School of Medicine at Mount Sinai, Queens Hospital Center, New York, USA; 3 Medicine, Icahn School of Medicine at Mount Sinai, Queens Hospital Center, New York, USA; 4 Geriatrics, New York University Langone Health, Bellevue Hospital Center, New York, USA

**Keywords:** coronavirus disease 19, sars-cov-2, obesity, bmi, older adults

## Abstract

Background and aim

Coronavirus disease 2019 (COVID-19) is known to cause a broad spectrum of illnesses. There is evidence that obesity-related conditions may increase the severity of COVID-19 disease, especially in those below the age of 60. However, there has been limited research on mortality rate based on body mass index (BMI) in the older adult population, defined as age over 65. The objective of this study was to characterize outcomes in older adults infected with COVID-19 based on BMI.

Study design and methods

It is a single-center retrospective cohort study of older adults with COVID-19 infection. The primary outcome was hospital mortality. Secondary outcomes assessed were oxygen requirements, need for mechanical ventilation, duration of mechanical ventilation, and hospital length of stay. Data were analyzed with the Student’s t-test, Fisher’s exact test, and multiple logistic regression analyses as appropriate.

Results

A total of 290 patients were included in this study. The mean age was 77.6 years. The median BMI was >30 kg/m2. The primary outcome of hospital mortality occurred in 49.7% of patients. BMI was not found to be a predictor of mortality. Age 75-79 and age ≥ 85 were associated with an increased risk of mortality (OR: 2.58; 95% CI: 1.15 - 5.79; OR: 3.17; 95% CI: 1.35 - 7.44, respectively). Patients with a BMI < 18.5, BMI 18.5 - 25, and age ≥ 85 were less likely to require mechanical ventilation (OR: 0.06; 95% CI: 0.00 - 0.83; OR:0.11; 95% CI: 0.02 - 0.64 and OR:0.28; 95% CI: 0.09 - 0.92, respectively). Past medical history was not associated with mortality.

Conclusion

In a cohort of older adults with COVID-19 disease, BMI was not an independent predictor of in-hospital mortality. Patients with BMI ≤ 25 and age ≥ 85 years were less likely to require mechanical ventilation.

## Introduction

The World Health Organization (WHO) declared coronavirus disease 19 (COVID-19) caused by severe acute respiratory syndrome coronavirus 2 (SARS-CoV-2), a global pandemic [1]. As of September 2020, more than 27 million cases have been reported worldwide, with an estimated mortality rate of 3.2% [2]. COVID-19 is known to cause a broad spectrum of diseases ranging from mild upper respiratory symptoms to acute respiratory distress syndrome (ARDS), hypercoagulability, cytokine storm, and hemophagocytic lymphohistiocytosis (HLH) [3].

Current literature suggests that obesity-related conditions may increase the severity of COVID-19, especially in those below the age of 60 [4]. However, there is limited data on mortality in obese patients with COVID-19 infection in the older adult population, defined as an age ≥ 65 years. Obesity, defined as a body mass index (BMI) >30 kg/m^2^, has increased worldwide over the last decade and will continue to do so without significant intervention. By 2025, the global rate of obesity is predicted to surpass 21% in women and reach 18% in men [5]. Obesity has numerous deleterious clinical effects such as limitation of diaphragmatic movement limiting ventilation, and impairment of host response to viral infection resulting in oxidative stress and subsequent reduction in cardiac function [6]. This study's primary objective was to evaluate the association between BMI and mortality in older adults infected with COVID-19.

## Materials and methods

Study design and patient population

We conducted a single-center retrospective cohort study of older adults with laboratory-confirmed COVID-19 infection via polymerase chain reaction (PCR) admitted to a community teaching hospital in New York City between February 1st, 2020 and April 30th, 2020. Patients were excluded from the study if they did not have a BMI documented or transferred to another acute care facility to continue care. The primary endpoint was hospital mortality. Secondary outcomes included supplemental oxygen requirements, need for mechanical ventilation, duration of mechanical ventilation, and hospital length of stay.

The study received approval from the Icahn School of Medicine at Mount Sinai and New York City Health and Hospitals/Queens Institutional Review Board with a waiver for informed consent (IRB 20-03186).

Outcomes and statistical analysis

BMI (kg/m^2^) was analyzed as a categorical variable. BMI was divided into six categories: BMI < 18.5, BMI 18.5-25.9, BMI 26-29.9, BMI 30-35.9, BMI 36-40, and BMI > 40. Patients were also grouped into four categories based on age: 70-75, 76-79, 80-84, and >85. The primary endpoint was hospital mortality. The secondary outcomes assessed were supplemental oxygen requirements, need for mechanical ventilation, duration of mechanical ventilation, and length of stay.

Statistical analyses were performed with SAS Studio (SAS Corporation, Cary, USA). Multiple logistic regression analysis was performed with hospital mortality and mechanical ventilation as the dependent variables and with BMI categories, age categories, and past medical history as the independent variables.

## Results

**Figure 1:**
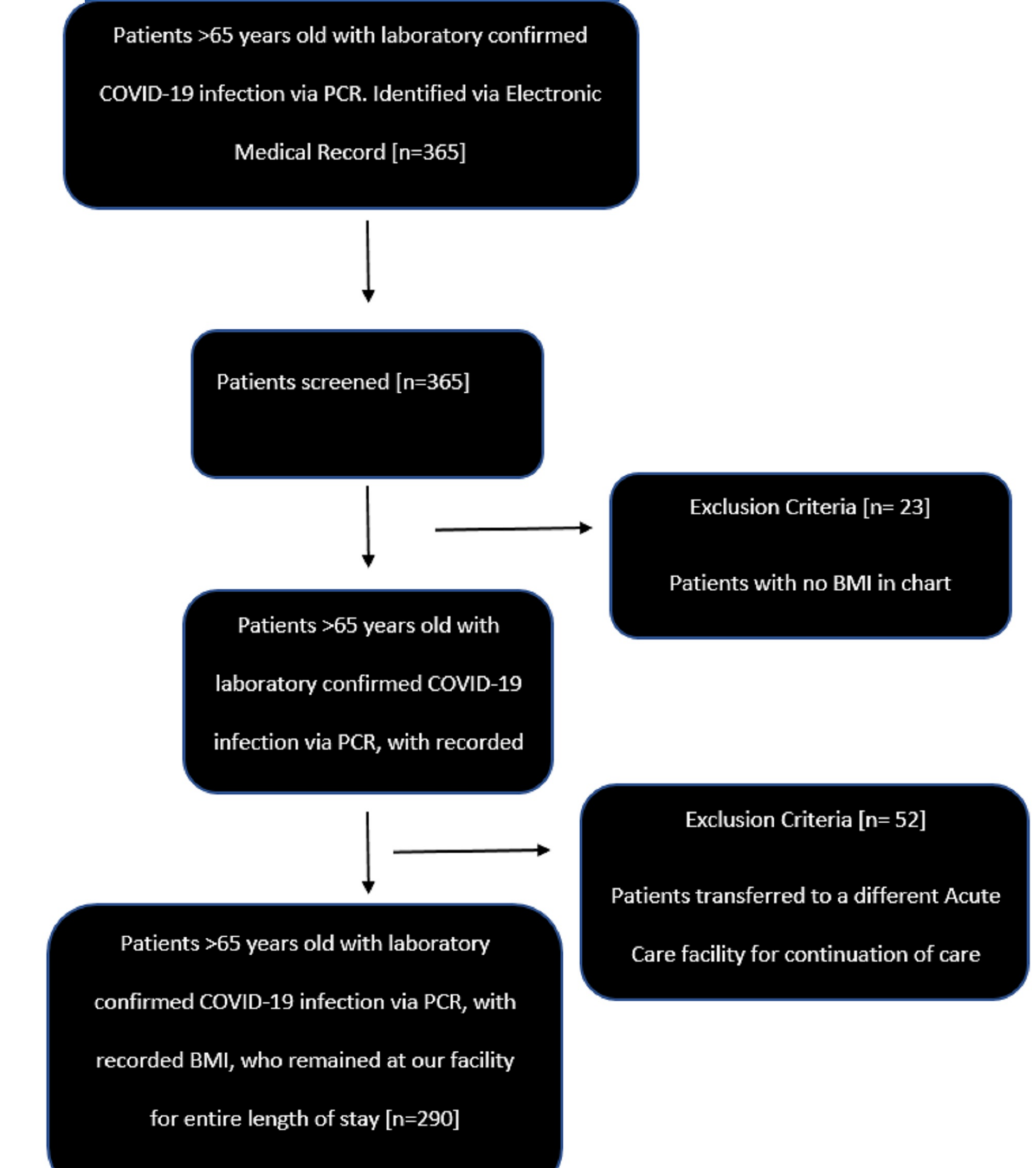
Patient inclusion in the study (consort diagram)

A total of 290 older adult patients with COVID-19 disease were included in this study (Figure 1). Detailed baseline characteristics, including the percentage of patients in each age and BMI category, are visualized in Table 1. Data on advanced directives were collected and showed that out of 290 patients, 23 (7.9%) had a do-not-resuscitate (DNR) order, 91 (31.4%) had a DNR/do-not-intubate (DNI) order, and 170 (58.6%) were full code. The mean hospital length of stay was 9 ± 9 days. Inpatient seven-day mortality was 31%, 14-day mortality was 43.8 %, and overall hospital mortality was 49.7% (see Table 2).

**Table 1:** Baseline characteristics (n=290)

Characteristic	N (%)
Age, mean ± SD	77.6 ± 8.3
Sex, male	150 (51.7%)
BMI < 18.5	20 (6.9%)
BMI 18.5 – 25.9	106 (36.6%)
BMI 26 – 29.9	75 (25.9%)
BMI 30 – 35.9	52 (17.9%)
BMI 36 – 40	17 (5.9%)
BMI > 40	20 (6.9%)
Past medical history	
Asthma	18 (6.2%)
Coronary artery disease	80 (27.6%)
Chronic obstructive pulmonary disease	19 (6.6%)
Diabetes mellitus	150 (51.7%)
End-stage renal disease	37 (12.8%)
Hypertension	236 (81.4%)
Obstructive sleep apnea	11 (3.8%)

**Table 2:** Patient outcomes

Outcome	N (%)
Required mechanical ventilation	62 (21.4%)
Hospital length of stay, days, mean ± SD	9 ± 9
7-day mortality	90 (31%)
14-day mortality	127 (43.8%)
Hospital mortality	144 (49.7%)

Multiple logistic regression analyses

Mortality

The first multiple logistic regression analysis was performed with mortality as the dependent variable and pre-defined BMI ranges, pre-defined age ranges, and past medical history as the independent variables. Two age ranges were found to be independent predictors for mortality: age 75-79 (OR: 2.58, 95% CI: 1.15 - 5.79) and age ≥ 85 years (OR: 3.17; 95% CI: 1.35 - 7.44). BMI, past medical history, and the other age ranges were not associated with risk for mortality (Table 3). 

**Table 3:** Multiple logistic regression analyses CAD - coronary artery disease; COPD - chronic obstructive pulmonary disease; DM - diabetes mellitus; ESRD - end-stage renal disease

Analysis	Independent variables	Regression coefficient	P-value	Odds ratio (95% CI)
Mortality	BMI < 18.5	-0.74	0.32	0.48 (0.11 – 2.06)
	BMI 18.5 – 25	-0.87	0.14	0.42 (0.13 – 1.31)
	BMI 26 – 29	-0.76	0.19	0.47 (0.15 – 1.46)
	BMI 30 – 35	-0.46	0.44	0.63 (0.20 – 2.02)
	BMI 36 – 40	0.00	1.00	1.00 (0.25 – 4.03)
	BMI > 40	-0.70	0.30	0.50 (0.13 – 1.85)
	Age 70 – 74	0.41	0.31	1.50 (0.69 – 3.30)
	Age 75 – 79	0.95	0.02	2.58 (1.15 – 5.79)
	Age 80 – 84	0.81	0.05	2.25 (1.00 -5.11)
	Age ≥ 85	1.15	0.01	3.17 (1.35 – 7.44]
	Asthma	-0.41	0.43	0.66 (0.24 – 1.83)
	CAD	-0.09	0.76	0.91 (0.52 – 1.62)
	COPD	1.31	0.02	3.69 (1.19 – 11.45)
	DM	0.34	0.20	1.41 (0.83 – 2.39)
	ESRD	-0.07	0.85	0.93 (0.44 – 1.98)
	Hypertension	-0.46	0.19	0.63 (0.32 -1.25)
Mechanical Ventilation	BMI < 18.5	-2.80	0.04	0.06 (0.00 – 0.83)
	BMI 18.5 – 25	-2.19	0.01	0.11 (0.02 – 0.64)
	BMI 26 – 29	-1.36	0.12	0.26 (0.05 – 1.44)
	BMI 30 – 35	-1.78	0.05	0.17 (0.03 – 1.01)
	BMI 36 – 40	-0.76	0.45	0.47 (0.07 – 3.36)
	BMI > 40	-0.30	0.75	0.74 (0.11 – 4.96)
	Age 70 – 74	-0.18	0.68	0.83 (0.35 – 1.99)
	Age 75 – 79	-0.38	0.41	0.68 (0.27 – 1.70)
	Age 80 – 84	-0.46	0.35	0.63 (0.24 – 1.66)
	Age ≥ 85	-1.27	0.04	0.28 (0.09 – 0.92)
	Asthma	-0.92	0.20	0.4 (0.10 – 1.61)
	CAD	-0.47	0.24	0.63 (0.29 – 1.36)
	COPD	0.12	0.86	1.13 (0.31 – 4.16)
	DM	0.65	0.06	1.91 (0.98 – 3.74)
	ESRD	-0.35	0.49	0.71 (0.26 – 1.90)
	Hypertension	-0.54	0.20	0.58 (0.25 – 1.34)

Mechanical Ventilation

A second multiple logistic regression analysis was performed with mechanical ventilation as the dependent variable and pre-defined BMI ranges, age ranges, and past medical history as the independent variables (Table 3). The independent predictors of mechanical ventilation were BMI < 18.5 (OR: 0.06; 95% CI: 0.00 - 0.83), BMI 18.5 - 25 (OR:0.11; 95% CI: 0.02 - 0.64), and age ≥ 85 years (OR:0.28; 95% CI: 0.09 - 0.92). 

## Discussion

In our retrospective cohort study of 290 older adults with COVID-19, BMI was not found to be an independent predictor of mortality. There is limited data on the association between BMI and COVID-19 related mortality in older adults. Current literature suggests an association between obesity and increased mortality in patients with COVID-19 pneumonia in the general population, especially in younger patients [7-9]. Baseline characteristics such as obesity, belonging to a minority population, older age, male gender, and comorbidities such as hypertension and diabetes have been described as risk factors for a poor prognosis [4, 7, 10-12].

In our cohort of patients, obesity was not found to be a risk factor for increased mortality or increased need for ventilation. However, a lower BMI ≤ 25 was found to be associated with a decreased need for mechanical ventilation. Two studies conducted in France reported a higher risk for developing more severe symptoms of COVID-19 in patients with a BMI ≥ 35 [4, 13].

The impact of obesity on lung function is likely multifactorial and is associated with both mechanical and inflammatory etiologies [6, 14]. Obesity creates a baseline inflammatory state that impairs the immune response to viral infections, has a major impact on respiratory function and diaphragmatic excursion, and increases the risk of developing lung disease, influenza, and bacterial pneumonia [6, 15]. Pellegrino et al. found that obesity alters the distribution of ventilation by airway narrowing and air trapping, increasing ventilation in the upper lobes, and contributing to ventilation inhomogeneity [16]. This airway narrowing and closure have also been found to exacerbate airway reactivity, affecting medication delivery, thus reducing the efficacy of inhaled medication [14]. Patients with obesity breathe at lower lung volumes as the reduction in functional residual capacity is proportional to the severity of obesity; thus, respiratory system mechanics are altered. Additionally, pulmonary function testing underestimates the effect of obesity on respiration as it uses forced maneuvers and deep breaths, which do not account for changes that occur during breathing at low functional residual capacity. Therefore, oscillometry may be a better measure of lung mechanics than spirometry in obese patients as it occurs during normal breathing [14].

One potential reason for the lack of statistical significance in our primary outcome is the use of BMI as a surrogate for obesity. Ceylan et al. suggest that while BMI is an important factor when it comes to assessing obesity, fat distribution may be more important. Fat distribution can be measured using a waist to hip circumference ratio (WHR); a high WHR measurement acts as a proxy for excessive intra-abdominal fat [17]. There are two main regional fat distribution patterns: central and peripheral. Central obesity is characterized by increased deposition of fat in the thorax, abdomen, and visceral organs. Peripheral obesity is characterized by the deposition of fat in the hips, thighs, limbs, and subcutaneous tissue [14, 18]. Collins et al. found that patients with a central (upper body fat distribution) had significantly lower functional vital capacity, forced expiratory volume in one second, and total lung capacity than those with peripheral obesity, indicating that central obesity is likely to have a more direct effect on pulmonary mechanics than peripheral obesity [19]. A greater fat deposit in the abdominal region generates a greater resistance to diaphragmatic contraction, hindering ventilatory mechanics [20]. Central obesity also has a greater impact on metabolic inflammation. Obesity is a chronic state of low-grade inflammation, the degree of which differs between subtypes of obesity. Visceral fat seen in central obesity is more metabolically active than subcutaneous fat seen in peripheral obesity [18]. Indeed, increased visceral fat mass is linked to the metabolic syndrome, and the metabolic syndrome has been linked to asthma and impaired lung function in both adolescents and adults [21]. Our study used BMI alone and did not account for weight distribution. Future studies can evaluate clinical outcomes utilizing weight distribution as a measure of obesity.

Unintentional weight loss has been associated with increased morbidity and mortality in older adults [22]. A European study revealed that high clinical frailty scores (CFS) were an independent predictor for higher mechanical ventilation risk in patients with COVID-19 [23]. However, our study found that lower BMI (≤ 18.5 and ≤ 25) was associated with a decreased need for mechanical ventilation. While our study focused on BMI, it did not evaluate for frailty. These findings imply that lower BMI alone is not an accurate predictor of outcome in patients with COVID-19. Frailty as a syndrome increases the susceptibility to stressors and should be studied further. 

Our patient population largely consisted of minorities, with the average demographic of patients being 9.8% White, 17.6% other, 27% Hispanic, and 32.7% Black, according to the 2020 New York City Health + Hospitals population health dashboard. Several studies in the United States and the United Kingdom have noted that COVID-19 has disproportionately affected minority-predominant populations [11, 12]. Estabragh et al. hypothesized that this may be explained by the fact that minority populations were more likely to have lower vitamin D levels, a worse cardioembolic profile, and were more likely to live in overcrowded households [11]. Mahajan et al. found a statistically significant association between numbers of African-American populations living in certain counties and the number of COVID 19 confirmed cases, confirmed death, and overall mortality. Positive correlations were also found between Asian-American populations and these factors, whereas negative correlations existed between Whites. Additional studies are required to examine further and confirm the associations between race and COVID-19 clinical outcomes [12].

There are several limitations to our study that we acknowledge. First, the relatively small sample size and retrospective design of our study limit the external validity of the results. Second, BMI is an imperfect measure of true obesity, and future studies should be conducted utilizing other surrogates of obesity. Furthermore, our study did not evaluate for frailty index, a predictor of higher mortality in older adults [24]. Lastly, we were unable to account for other confounders that may have impacted patient mortality, such as the various treatment modalities for COVID-19.

## Conclusions

In a single center retrospective cohort analysis of older adults with COVID-19 infection, BMI was not found to be an independent predictor of mortality. Age 75-79 and age ≥ 85 years were found to be a positive predictor of mortality. BMI < 18.5, BMI between 18.5 and 25.9, and age > 85 years were less likely to require mechanical ventilation. Additional studies are needed to assess the association between age, obesity, frailty, and clinical outcomes in older adults with COVID-19 disease.

## References

[1] (2019). World Health Organization: Coronavirus disease 2019 [COVID-19] Situation Report - 51. https://www.who.int/docs/default-source/coronaviruse/situation-reports/20200311-sitrep-51-covid-19.pdf.

[2] (7 September 2020). World Health Organization: Weekly Epidemiological Update Coronavirus disease 2019 [COVID-19]. https://www.who.int/docs/default-source/coronaviruse/situation-reports/20200907-weekly-epi-update-4.pdf.

[3] Sohrabia C (2020). World Health Organization declares global emergency: A review of the 2019 novel coronavirus (COVID-19). Int J Surg.

[4] Caussy C, Alsafi Z, O'Neill N (2020). Prevalence of obesity among adult inpatients with COVID-19 in France. Lancet Diabetes Endocrinol.

[5] Leach R, Powis J, Baur LA (2020). Clinical care for obesity: a preliminary survey of sixty-eight countries. Clinical Obesity.

[6] Kass D, Duggal P, Cingolani O (2020). Obesity could shift severe COVID-19 disease to younger ages. Lancet.

[7] Palaiodimos L, Kokkinidis DG, Weijia L (2020). Severe obesity, increasing age and male sex are independently associated with worse in-hospital outcomes, and higher in-hospital mortality, in a cohort of patients with COVID-19 in the Bronx, New York. Metabolism.

[8] Busetto L, Bettini S, Fabris R (2020). Obesity and COVID- 19: an Italian snapshot. Obesity.

[9] Hajifathalian K, Kumar S, Newberry C (2020). Obesity is associated with worse outcomes in COVID- 19: analysis of early data from New York City. Obesity.

[10] Zhao H, Huang Y, Huang Y (2020). Mortality in Older Patients with Covid-19. J Am Geriatr Soc.

[11] Estabragh Z, McCracken C, Bethell MS (2020). Greater risk of severe COVID-19 in Black, Asian and Minority Ethnic populations is not explained by cardiometabolic, socioeconomic or behavioural factors, or by 25(OH)-vitamin D status: study of 1326 cases from the UK Biobank. J Public Health.

[12] Mahajan U, Larkins-Pettigrew M (2020). Racial demographics and COVID-19 confirmed cases and deaths: a correlational analysis of 2886 US counties. J Public Health.

[13] Simonnet A, Chetboun M, Poissy J (2020). High prevalence of obesity in severe acute respiratory syndrome coronavirus-2 [SARS-CoV-2] requiring invasive mechanical ventilation. Obesity.

[14] Dixon E, Peters U (2018). The effect of obesity on lung function. Expert Rev Respir Med.

[15] Fezeu L, Julia C, Henegar A (2011). Obesity is associated with higher risk of intensive care unit admission and death in influenza A (H1N1) patients: a systematic review and meta-analysis. Obesity Reviews.

[16] Pellegrino R, Gobbi A, Antonelli A (2014). Ventilation heterogeneity in obesity. J Appl Physiol.

[17] Ceylan E, Çömlekçi A, Akkoçlu A (2009). The effects of body fat distribution on pulmonary function tests in the overweight and obese. South Med J.

[18] Kang SM, Yoon JW, Ahn HY (2011). Android fat depot is more closely associated with metabolic syndrome than abdominal visceral fat in elderly people. PLoS ONE.

[19] Collins C, Hoberty D, Walker F, Fletcher EC, Peiris AN (1995). The effect of body fat distribution on pulmonary function tests. Chest.

[20] Melo L, da Silva MAM, do Nascimento Calles AC (2014). Obesity and lung function: a systematic review. Einstein.

[21] Serafino-Agrusa L, Spatafora M, Scichilone N (2015). Asthma and metabolic syndrome: current knowledge and future perspectives. World J Clin Cases.

[22] Stefani F, Pietraroia P, Faria-Neto J (2018). Observational evidence for unintentional weight loss in all-cause mortality and major cardiovascular events: a systematic review and meta-analysis. Sci Rep.

[23] Labenz C, Kremer W, Schattenberg J (2020). Clinical Frailty Scale for risk stratification in patients with SARS-CoV-2 infection. J Investig Med.

[24] Kojima G, Iliffe S, Walters K (2018). Frailty index as a predictor of mortality: a systematic review and meta-analysis. Age Ageing.

